# Comparative study of laboratory and portable spot permeability measurements in bioturbated carbonate strata

**DOI:** 10.1038/s41598-025-00697-w

**Published:** 2025-05-07

**Authors:** Ayman Al-Lehyani, Hassan A. Eltom, Abdullatif Al-Shuhail, Eassa Abdullah, Ammar El-Husseiny

**Affiliations:** 1 Geosciences Department, College of Petroleum Engineering Geosciences, King Fahd University of Petroleum & Minerals, Dhahran, Saudi Arabia; 2 Center for Integrative Petroleum Research, College of Petroleum Engineering Geosciences, King Fahd University of Petroleum & Minerals, Dhahran, Saudi Arabia

**Keywords:** Spot permeability, TinyPerm 3, AutoScan, Bland-Altman plot, Carbonate, Outcrop, Hanifa formation, Ulayyah member, Saudi Arabia, New England research, Geology, Sedimentology, Petrology, Carbon capture and storage, Hydrogen storage, Crude oil, Natural gas, Petrol, Hydrogen energy

## Abstract

In bioturbated strata, defining and obtaining a representative elementary volume for permeability measurements presents significant challenges. To address this, spot permeability is often used as an alternative method to estimate the bulk permeability of these complex strata. However, laboratory measurements of spot permeability require steady-state nitrogen gas injection and careful sample preparation including slab cutting which makes the process both time-consuming and resource-intensive. Alternatively, field measurements using portable devices offer a more cost-effective, straightforward, and real-time approach for assessing permeability in situ. To evaluate the accuracy of portable devices in measuring spot permeability in bioturbated strata, we compare and contrast permeability measurements on the same rock samples (from the Hanifa Formation in central Saudi Arabia) using two different techniques: the sophisticated laboratory-based AutoScan system and the portable TinyPerm 3 field device both from New England Research (NER). Two sets of permeability data comprising 64 measurements were obtained and analyzed graphically using Whisker Box Plots, Bland-Altman Plot, Curve Matching, and Cross Plots, as well as statistically through correlation analysis and analysis of variance (ANOVA). The graphical analysis revealed no substantial difference in permeability measured by the two methods, a finding further supported by ANOVA results, which indicated no statistically significant difference (P-value > 0.05). Additionally, the correlation plot between the permeability measurements from the two methods yielded a straight line with an R$^2$ value greater than 0.9, demonstrating a strong agreement. This study highlights the potential of using portable handheld permeameters to measure spot permeability of bioturbated strata in the field, offering a cost-effective, accurate, and real-time alternative to laboratory-based techniques in estimating their bulk permeability.

## Introduction

In the stratigraphic record, considerable intervals of sedimentary strata exhibit signs of bioturbation^1–6^. This process, bioturbation, encompasses a range of organism-induced activities, such as sediment compaction, sorting, and pack filling, leading to substantial alterations in both the chemical and physical properties of sediments^7–9^. Importantly, these bioturbated strata represent key stratigraphic intervals, often serving as sources for significant natural resources such as hydrocarbons and water^6,10–18^.

Bioturbation significantly influences the reservoir and aquifer quality of sedimentary strata, with the potential for both enhancement and deterioration^15,19–23^. Enhancement occurs when burrow fillings are porous and permeable, forming a network within the sedimentary strata. This network establishes permeability pathways that enhance storage and flow capacity^24^. On the other hand, reservoir and aquifer quality deteriorates if the burrows, or portions of them, are filled with tight, less permeable sediments^23,25^. This dual impact of bioturbation underscores its complex role in shaping the characteristics of bioturbated strata, particularly their porosity and permeability.

While laboratory measurements of porosity in bioturbated strata are relatively straightforward, accurately assessing their permeability is considerably more challenging^26–28^. This challenge primarily stems from the complex and heterogeneous nature of bioturbated strata. These strata frequently exhibit complicated burrow morphologies that control the distribution of permeable sediments within the rock matrix^22^. Consequently, conventional methods for measuring permeability, which are effective in more homogeneous sedimentary layers, may not provide accurate or representative results when applied to bioturbated strata^18,20^. This is largely due to the inherent difficulty in acquiring a correctly representative elementary volume of these strata, given their irregular and varied structural features^29,30^. This situation underscores the necessity of developing specialized techniques or adapting existing methodologies to evaluate the permeability of these unique biogenetic structures in sedimentary strata effectively and accurately.

Spot permeability measurements (permeability measurements at one spot on the rock sample surface compared to core flooding measurements) have emerged as a solution to address the challenges in estimating the bulk permeability of bioturbated strata. For example, the research conducted by Gingras et al.^26^ and Baniak et al.^15^ introduced concepts such as dual-permeability fluid flow media (D-PermFFM) and dual-porosity fluid flow media (D-PorFFM). These terms describe bioturbated strata where permeability contrasts are markedly different, with D-PermFFM exhibiting contrasts greater than three orders of magnitude and D-PorFFM showing contrasts less than two orders of magnitude^15^. These studies propose that the bulk permeability in bioturbated reservoirs, inferred from spot permeability measurements, varies depending on the type of fluid flow media (D-PermFFM or D-PorFFM) and the burrow percentage. For example, in a D-PermFFM scenario, the bulk permeability can be represented by the geometric mean of spot permeability measurements when the burrow percentage ranges from 25% to 65% and by the arithmetic mean for burrow percentage ranging from 65% to 80%^15^. In scenarios characterized by D-PorFFM, it is recommended to use a geometric mean for burrow percentage between 10% and 50% and a harmonic mean for burrow percentage between 50% and 80%^15^. These methodologies reflect an adaptive approach to measuring permeability in bioturbated strata, accommodating the unique structural complexities of these strata.

**Fig. 1:**
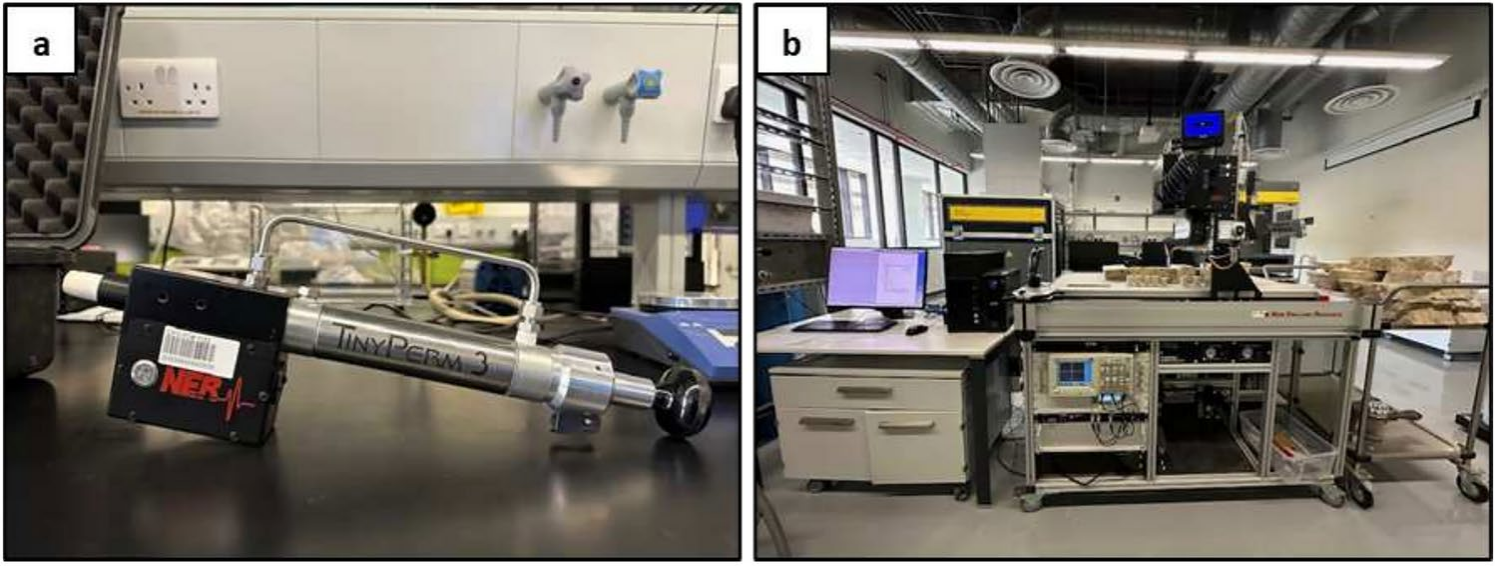
(**a**) The Portable TinyPerm 3 system, usually used to measure permeabilities in the field, and (**b**) the AutoScan system, used to measure permeabilities in the lab.

The significant challenge in conducting spot permeability measurements lies in the necessity for a sophisticated laboratory setup, which encompasses a range of complex and specialized components. This methodology requires not only advanced laboratory equipment but also a rigorously controlled environment, specialized technical expertise, and frequently a customized arrangement for each specific study. In response to these challenges and with the goal of finding a more accessible and user-friendly method, this study investigated the use of a portable system for the measurement of spot permeability in bioturbated strata. This portable approach offers the potential for more flexible and less resource-intensive measurements when compared to traditional laboratory setups. To assess the effectiveness of this portable system, this study employs advanced statistical analysis to compare spot permeability measurements obtained from both the conventional laboratory system represented by AutoScan and a portable system represented by TinyPerm 3 both from New England Research (NER) (Fig. 1). This comparative analysis aims to establish the validity and reliability of the portable system as a feasible alternative for spot permeability measurements in bioturbated strata. It highlights the potential of using portable handheld permeameters to measure spot permeability of bioturbated strata in the field, offering a quick and real-time estimation of their bulk permeability using averaging techniques as explained in Baniak et al.^31^. This will allow reservoir modelers to build better and more representative models of bioturbated reservoirs.

## Methodology

### Portable spot permeability measurements

Portable permeability measurements were conducted using the NER TinyPerm 3 system (Fig. 1a). This system (weighing 1.2 kg) is a handheld air permeameter that is used to infer the permeability of the rock matrix or effective fracture apertures from the gas flow rate, gas pressure, and permeameter probe diameter. It works by pressing a rubber nozzle against the rock sample and extracting air using a single-stroke syringe. A microcontroller unit keeps track of the syringe’s volume, and the transient vacuum pulse generated on the sample surface as air is removed. Measurements are automatically recorded and cataloged on an Android device, which also displays in real time a graph of pressure and volume data over time. The gathered data, which include permeability measurements, GPS coordinates, serial number, date, barometric pressure, ambient temperature, and humidity, are stored in the data files. These files can be exported to desktop computers for further analysis. The permeability measurement range for the TinyPerm 3 for intact rocks is from 1 millidarcy to 10 Darcy.

### Laboratory spot permeability measurement

For the laboratory measurements, the NER AutoScan system (Fig. 1b) was utilized. It is a fully automated nondestructive measurement system that uses different probes to measure spot permeability, ultrasonic velocity (compressional and shear), electrical resistivity, hardness, and composition using Fourier transform infrared (FTIR) spectroscopy. The system operates via a connected workstation that allows the user to define a 2D grid of measurement points. For spot permeability measurements, this system uses a 4 mm permeability probe and employs a steady-state gas injection technique to assess a broad spectrum of permeabilities, ranging from 0.01 millidarcy (mD) to 3 Darcy^32^.

Prior to measurement, the AutoScan’s gas injection probe was calibrated according to the manufacturer’s guidelines using reference materials to ensure baseline accuracy. The rock slab was prepared with a flat surface to ensure consistent surface contact. Then, the rock slab was mounted on the measurement platform, and the zero coordinate for the probe was defined for precise positioning. The coordinates for all the measurement points on the rock slab (relative to the probe zero position) were uploaded to the system to start the measurements. The system starts the measurements by moving the probe to the desired coordinate and presses it gently but firmly onto the rock surface to establish an airtight seal. A nitrogen gas flow is introduced under controlled pressure, and the resultant flow rate was recorded once steady-state flow conditions were reached. The system’s built-in algorithms calculate permeability values based on Darcy’s Law and the required corrections for gas slippage and inertial flow effect, taking into account gas viscosity, flow rate, injection pressure, and probe geometry^33^. Each point is measured three times to account for variability, and the mean of the three readings was used across all sessions.

### Samples for bioturbated strata

**Fig. 2:**
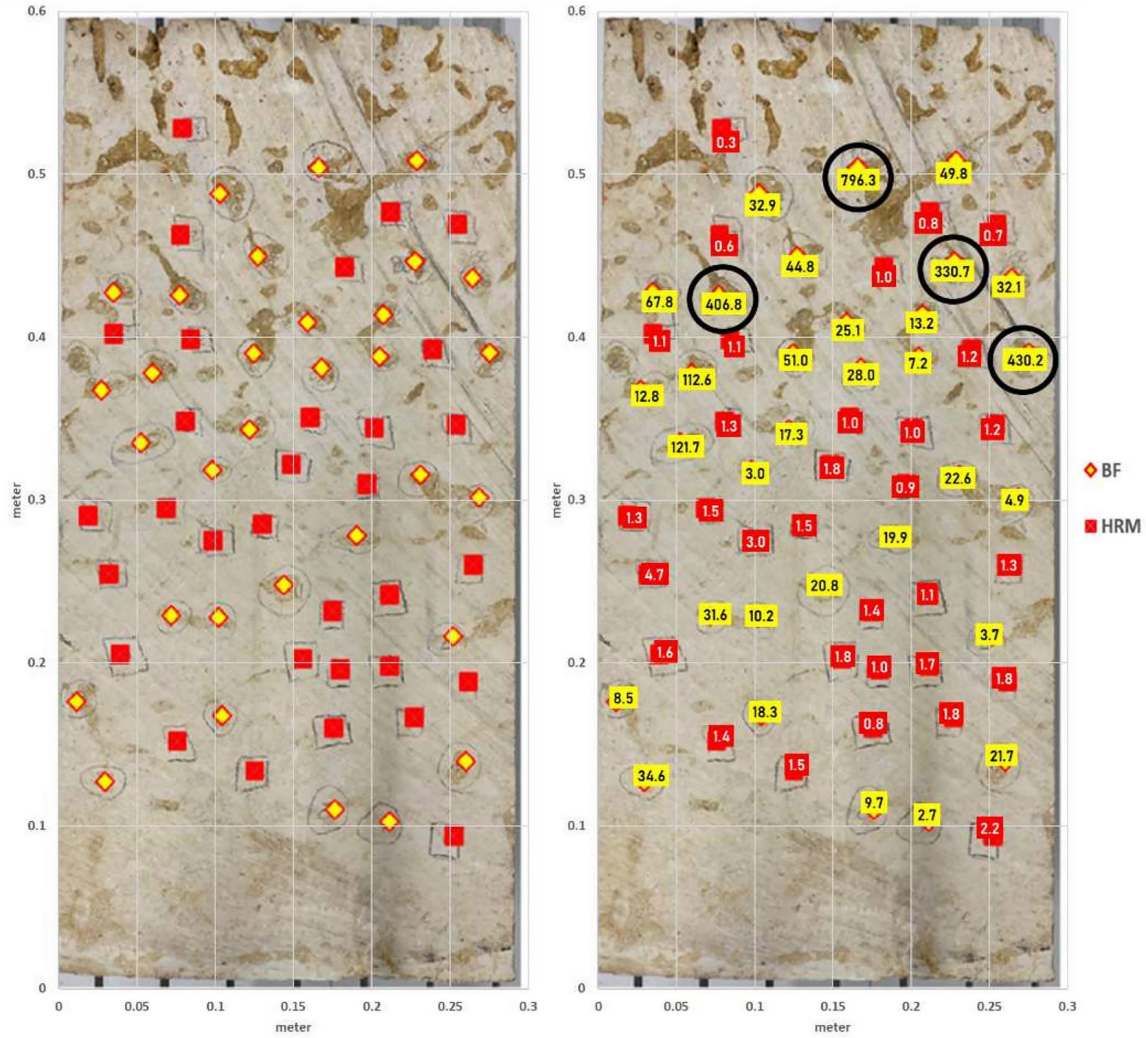
Images of the rock slab with locations of the permeability measurements in host rock matrix (HRM) indicated by red squares and in burrow fill (BF) indicated by yellow diamond. The right image shows the values of these measurements in HRM (red) and in BF (yellow). The values in black circles are the BF permeability measurements that are much higher than the other readings in the same group.

To assess the spot permeability of the bioturbated strata, a boulder-sized rock sample was collected from the Hanifa Formation outcrop (the upper most bioturbated strata of the Ulayyah Member), which is located at Jabal Abakkayn northwest of Riyadh, Saudi Arabia^24,29,30,34^. This boulder was carefully cut into a rectangular prism measuring 58 cm by 30 cm at the base and 10 cm in height. On the surface of this prepared slab, a total of 64 points were designated for spot permeability measurements. The permeability at each of these 64 points was measured using two different systems: the TinyPerm 3, a portable device designed for in-field permeability assessment, and the AutoScan system, a more sophisticated laboratory-based method for measuring permeability. These points were strategically selected to represent both the burrow fill (BF) and the surrounding host rock matrix (HRM), with half of the points allocated to each (Fig.2).

**Fig. 3:**
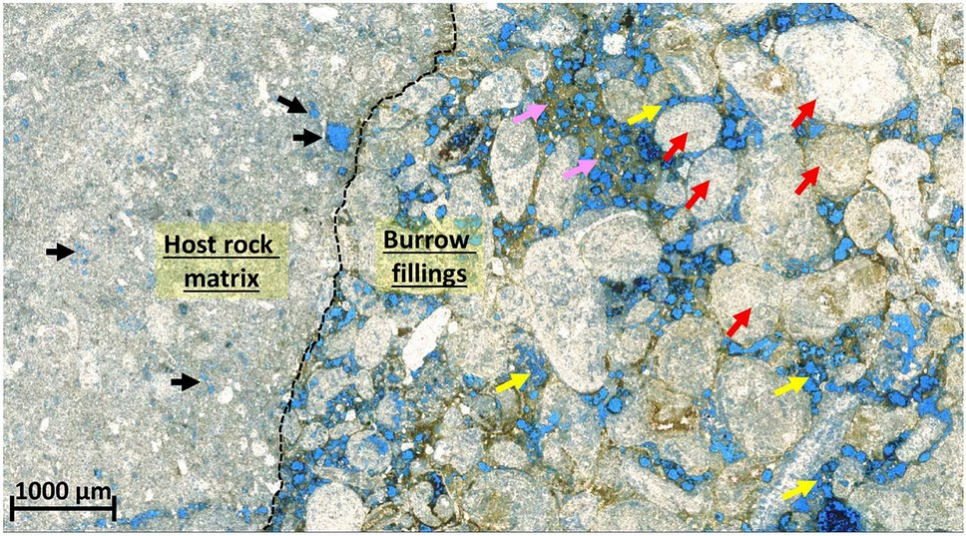
Photomicrograph of a thin section from the studied bioturbated strata, highlighting the two major components of the rock texture-host rock matrix (HRM) and burrow fillings (BF)-each with distinct textures, compositions, and pore types. The HRM is characterized by a mud-dominated texture with isolated moldic pores (black arrows). In contrast, the BF exhibits a grain-dominated texture with compacted grains (red arrows), primarily interparticle pores (yellow arrows), and a significant amount of muddy matrix with dissolved dolomite rhombs (pink arrows).

Both the BF and the HRM are major components of the rock samples, though they vary in proportion. The BF comprises approximately 20% of the rock, while the HRM makes up the remaining 80%. Figure 3 presents a photomicrograph of a thin section from the studied bioturbated strata, offering a detailed visual comparison of these components. The HRM is characterized by a mud-dominated texture, whereas the BF exhibits a grain-dominated texture. These textural differences are further highlighted by the distinct pore systems within each component. Moldic pores are predominantly found within the HRM, while interparticle pores are more prevalent in the BF. The variation in pore types between the HRM and BF plays a critical role in determining pore connectivity, which directly impacts permeability. This permeability contrast contributes to the development of a dual porosity system and leads to a bimodal distribution of pores within the bioturbated strata as suggested by previous studies^24,29,30^.

The data collected from both instruments (TinyPerm 3 and AutoScan) were analyzed and compared across two distinct groups of slabbed rock samples: burrow fill and host rock matrix. This comparison was conducted using both graphical and statistical methods to ensure a comprehensive understanding of the variations in permeability readings when using these two different methods. This dual approach enhanced the accuracy of the interpretation of the data.

### Graphical analysis

In our analysis, the graphical method was employed to observe the similarities and differences in permeability measurements between the burrow fill and host rock matrix groups when TinyPerm 3 and AutoScan were used to measure permeability. This approach comprises several techniques:

1. Whisker Box Plots: These plots were used to visually represent the distribution of the permeability measurements. They provided a clear view of the range, median, and potential outliers in the data, thereby offering an initial comparison of the variability within each group.
2. Curve Matching: This technique involved overlaying the permeability curves from the two groups. By comparing these curves, we could assess the degree of similarity in the permeability trends, providing a direct visual comparison of the data patterns.
3. Cross Plot: The cross-plot method was applied to plot the permeability measurements of one group against the other. This allowed us to observe any direct correlation or discrepancies between the two sets of measurements, offering insights into their relative behavior.
4. Bland-Altman Plot: The Bland-Altman plot was particularly valuable for evaluating the agreement between the two measurement methods. By plotting the differences against the averages of the two sets of measurements, we could visually assess the consistency and identify any systematic bias or trends in the data.

Complementing these graphical methods, the line fitting approach, specifically linear regression, was utilized to model the relationship between the measurements obtained from the two instruments. The slope and intercept of the fitted line provided insights into the alignment of the measurements and potential systematic biases. Additionally, the examination of residuals was crucial in identifying any individual anomalies or deviations in the measurements.

### Statistical analysis

To systematically explore the similarities and differences in permeability readings obtained from TinyPerm 3 and AutoScan, our initial step involved a thorough examination of the descriptive statistics for the permeability data from each system. Key statistical parameters such as the mean, minimum, maximum, range, standard deviation, and variance were utilized to preliminarily assess the similarities and differences in the measurements. Furthermore, to rigorously determine whether there were statistically significant differences in the permeability measurements between TinyPerm 3 and AutoScan, we employed one-way analysis of variance (ANOVA). This statistical test was crucial in evaluating the variance between the two systems and establishing the significance of any observed differences.

Additionally, to understand the degree of correlation between the permeability data measured by TinyPerm 3 and those measured by AutoScan, regression analysis was conducted. This approach allowed us to quantify the relationship between the datasets and provided insights into how closely the measurements from the two systems aligned. In all our statistical analyses, the significance level (P-value) was set at 0.05. This threshold was used to determine the statistical significance of our results, ensuring that any conclusions drawn were both reliable and valid in the context of comparing the performance and output of the TinyPerm 3 and AutoScan systems in measuring permeability.

## Results

### Graphical comparison of permeability: TinyPerm 3 vs AutoScan

**Fig. 4:**
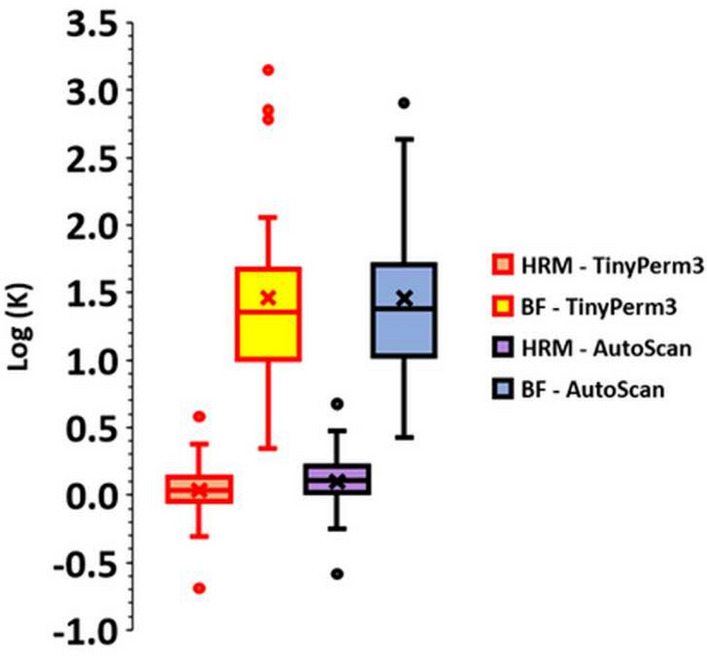
Whisker and box plot comparing the logarithm of permeability measurements in host rock matrix (HRM) and burrow fillings (BF) The data is presented on a logarithmic scale, illustrating the permeability measurements obtained using TinyPerm 3 and AutoScan. Note that with few exceptions, the measurements across both groups are predominantly within a similar range.

Figure 4 shows whisker box plots of the logarithm of the permeability measurements in the host rock matrix and burrow fillings measured by the TinyPerm 3 and AutoScan methods. While the two datasets show similar ranges, the TinyPerm 3 measurements exhibit slightly lower values than those of AutoScan.

**Fig. 5:**
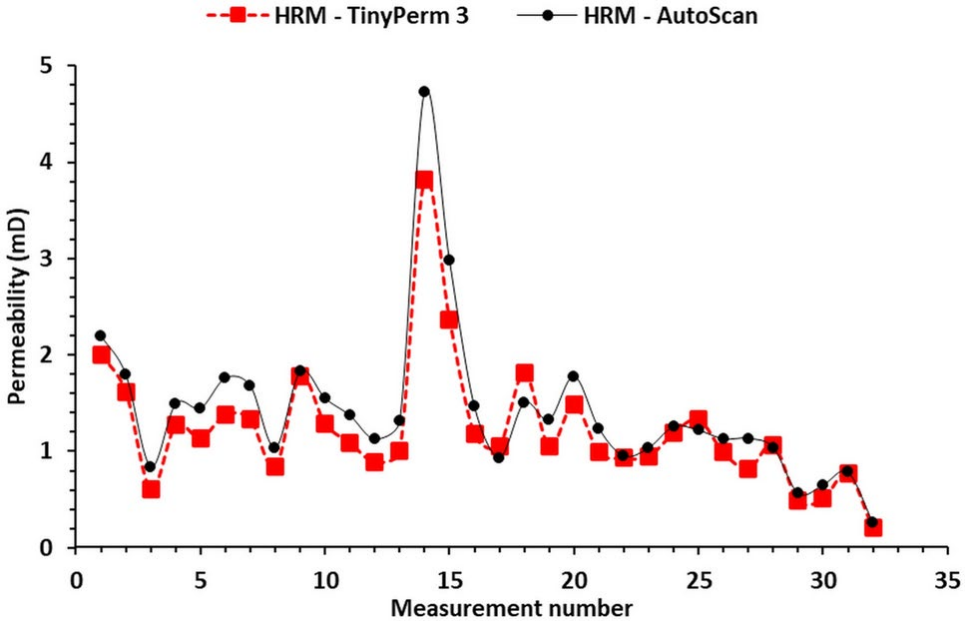
Plot of permeability measurements against measurement number in the slabbed samples. The graphs display permeability data for the host rock matrix (HRM) obtained using two distinct methods: TinyPerm 3 and AutoScan. The plot indicates that, with minimal variance, the permeability measurements from HRM measured by each method fall within a comparable range.

**Fig. 6:**
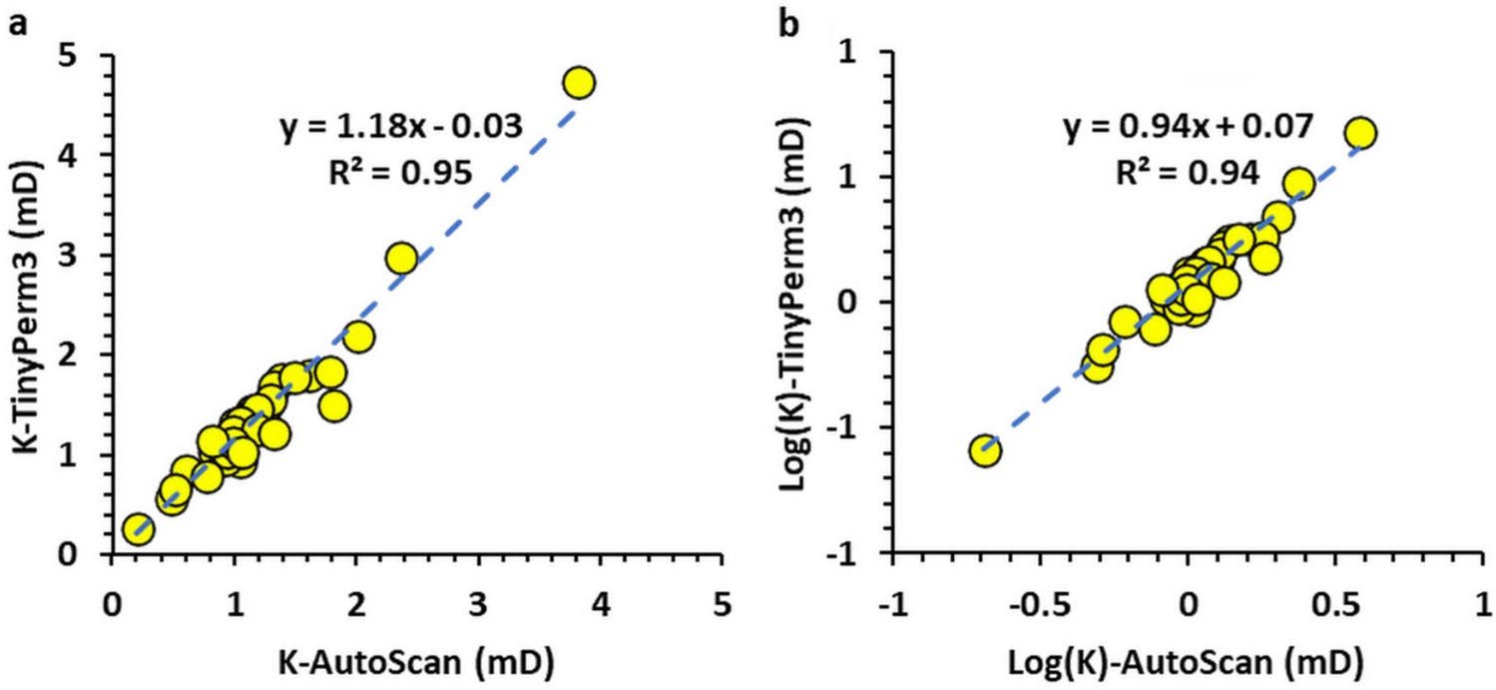
(**a**) Plot of the host rock matrix permeabilities (K) measured by the TinyPerm 3 and AutoScan showing a good correlation as evidenced by a slope and intercept values of 1.18 and 0.03 of the best-fit line (dashed), respectively and (**b**) same plot but for log(K)-Log(K) values showing a good correlation with a slope and intercept values of 0.94 and 0.07 of the best-fit line (dashed), respectively.

Figure 5 shows a plot of the 33 measured permeabilities of the host rock matrix in the studied sample using both the AutoScan and portable TinyPerm 3 permeameters. Most measurements show general agreement between the two permeabilities, with the AutoScan permeability exhibiting slightly greater values than those of the TinyPerm 3. Furthermore, plotting the permeability measurements of the host rock matrix measured by TinyPerm 3 and AutoScan on a linear-linear scale generally shows good agreement, as evidenced by slopes and intercepts of approximately 1.18 and 0.03, respectively, of the best-fit line (Figure 6a).

Figure 6b is a log-log plot of the two permeabilities, which also shows good agreement between the two permeabilities, with slopes and intercepts of approximately 0.94 and 0.07, respectively, for the best-fit line. Therefore, the AutoScan permeability and the portable TinyPerm 3 measurements are closely correlated in the host rock of the Hanifa Formation.

**Fig. 7:**
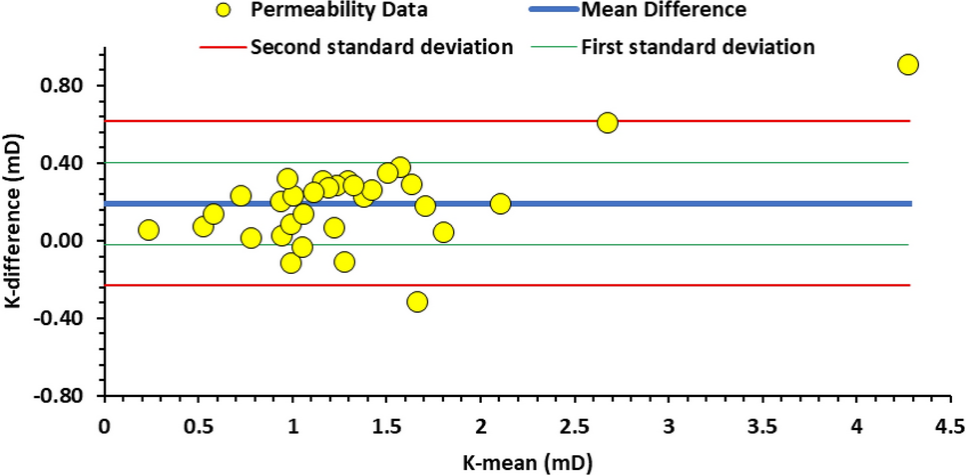
Bland-Altman plot for the host rock matrix for the AutoScan the TinyPerm 3 permeability measurements showing good agreement except for few higher permeability values.

Figure 7 shows a Bland-Altman plot for the host rock matrix for the AutoScan TinyPerm 3 permeability measurements. The mean difference (the blue solid line) is close to zero (0.18 mD), indicating minimal bias between the two methods. Most data points are within the limits of agreement (red lines), suggesting that the differences between measurements from both devices are consistent and within an acceptable range. However, there is a slight trend where differences increase as the mean of the measurements increases. This indicates that the two methods may not agree at higher permeability values. Overall, the plot suggests good agreement between the two instruments.

**Fig. 8:**
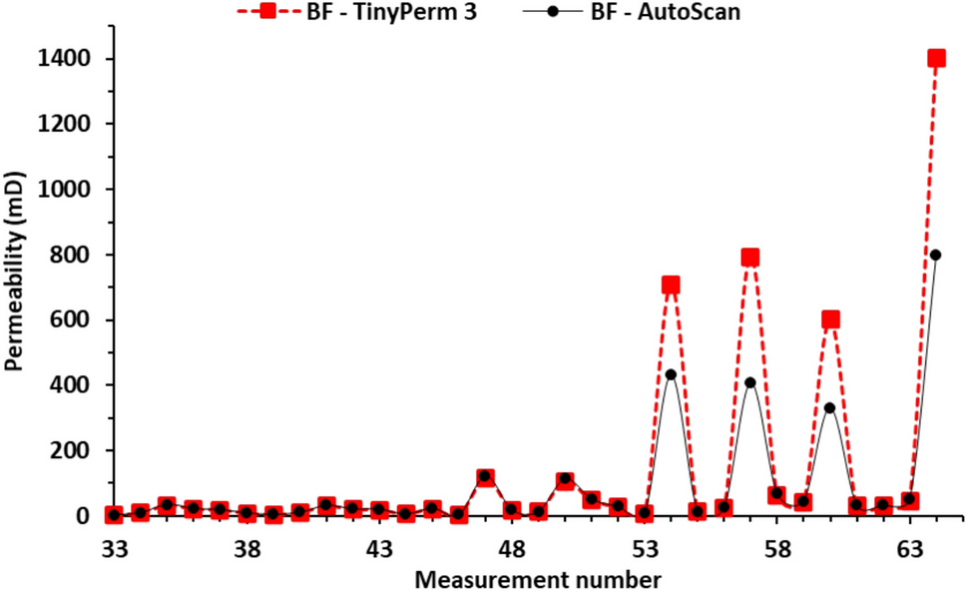
Plot of permeability measurements against measurement number in the slabbed samples. The graphs display permeability data for burrow fillings (BF) obtained using the TinyPerm 3 and AutoScan methods. The plot indicates that most permeability measurements from BF measured by the two methods fall within a comparable range.

**Fig. 9:**
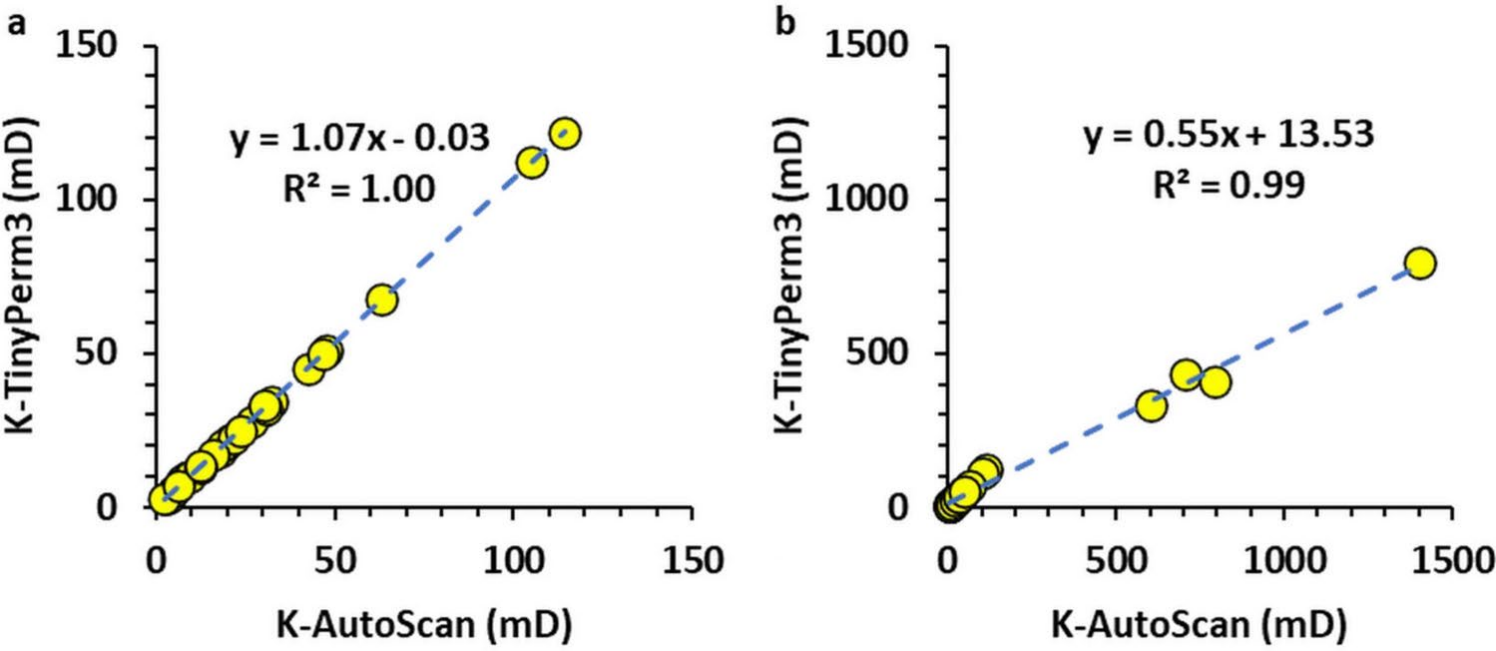
(**a**) plot of the burrow filling permeabilities measured by the TinyPerm 3 and AutoScan (excluding high-permeability measurement). The two permeabilities show a good correlation as evidenced by a slope and intercept values of 1.07 and -0.03 of the best-fit line (dashed), respectively. (**b**) The same plot but including high-permeability measurement. The two permeabilities show less correlation due to including the 4 different high-permeability values.

Figure 8 shows a plot of the measured AutoScan and TinyPerm 3 data for the burrow filling of the studied sample using a linear scale. The permeabilities show a bimodal distribution. Most (n=29) data points have a mean permeability of approximately 30 mD, while the remaining 4 data points have a considerably larger mean permeability of approximately 700 mD. The low-permeability measurements were almost identical for AutoScan and TinyPerm 3. On the other hand, the AutoScan and TinyPerm 3 data points have distinctly variable means of 491 mD and 877 mD, respectively. Curiously, one of the 4 different data points (no. 64) is quite different from the other 3 samples, with AutoScan and TinyPerm 3 values of 796 mD and 1404 mD, respectively. Plots of the permeabilities on a linear-linear scale show best-fit lines with slopes of approximately 1.07 when only the low-permeability values are fitted (Fig. 9a) and 0.55 when all the values are included in the fitting (Figure 9b).

**Fig. 10:**
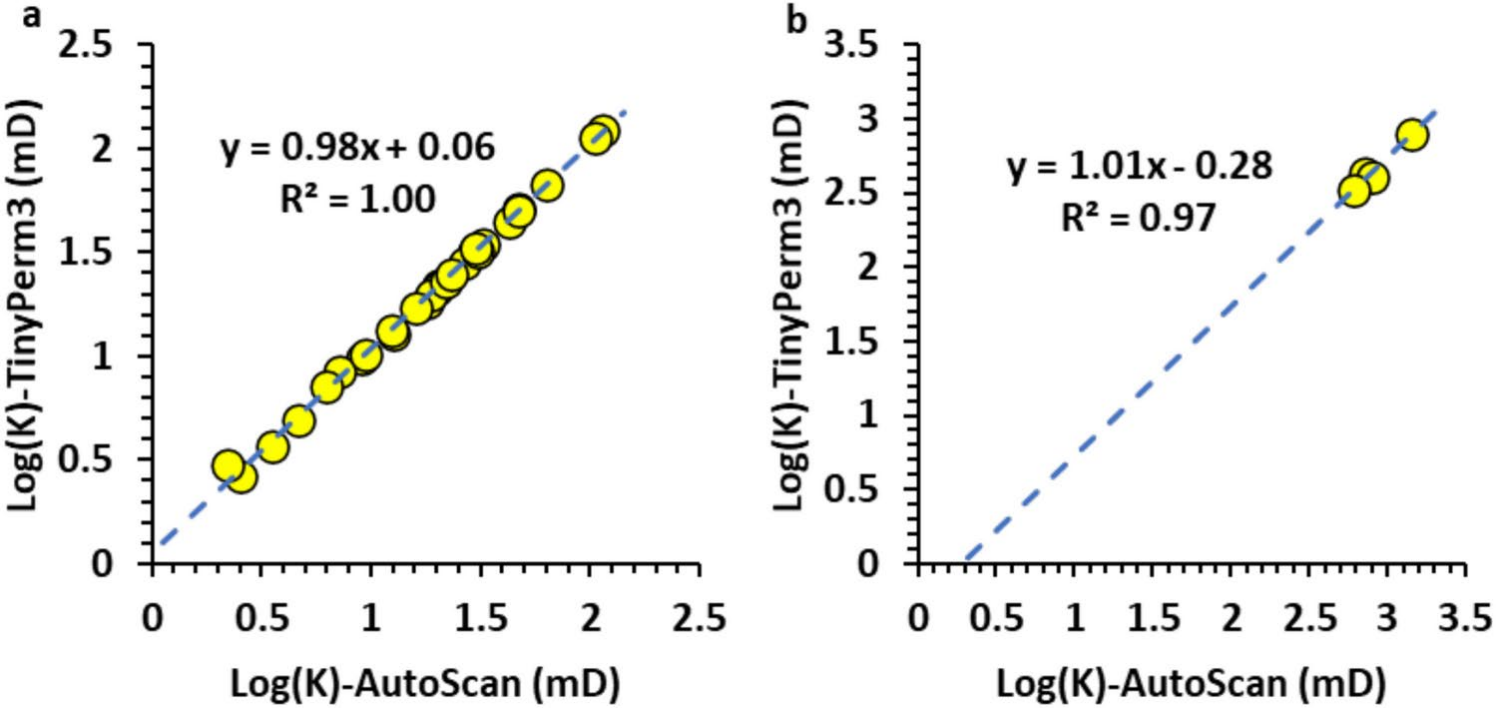
(**a**) Log-log plot of the low-permeability values showing an excellent agreement between the two methods. (**b**) Log-log plot of the high-permeability values in the burrow filling showing an overestimation of the TinyPerm 3 method as evidenced by an intercept of -0.28.

Plotting the two permeabilities on a log-log scale further exemplifies the agreement between the AutoScan and the TinyPerm 3 measurements. In particular, the best-fit line for the low-permeability samples shows a slope and intercept of 0.98 and 0.06 (Fig.10a), respectively. The proximity of the best-fit line slope to 1.0 indicates an excellent positive correlation between the two permeabilities, while an intercept close to 0.0 indicates the high accuracy of the TinyPerm 3 measurement at low permeabilities. Furthermore, the best-fit line to the 4 high-permeability data points shows a slope and intercept of 1.01 and -0.28, respectively (Fig 10b). Similar to the low-permeability data points, the proximity of the best-fit line slope to 1.0 indicates an excellent positive correlation between the two permeabilities. However, an intercept of -0.28 for the best-fit line on a log-log plot indicates that the TinyPerm 3 measurement overestimates the AutoScan permeability by approximately 100.28 = 1.9 times. This overestimation factor is close to that observed in the AutoScan and TinyPerm 3 means for these samples (i.e., 877/491 = 1.79 times).

**Fig. 11:**
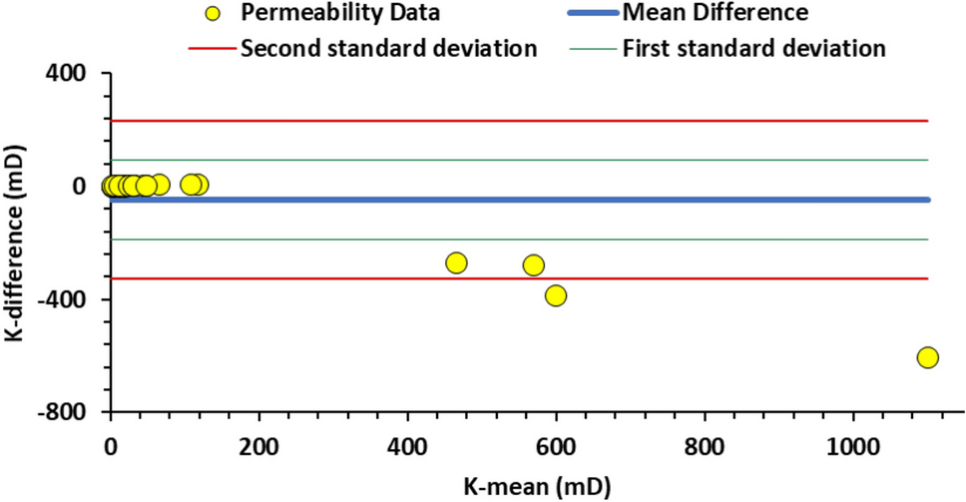
Bland-Altman plot for the burrow filling permeability measurements. This plot suggests that while the two techniques have generally good agreement, they measure different values at high permeabilities.

Figure 11 shows a Bland-Altman plot for the burrow filling permeability measurements. As in the host rock matrix case, the mean difference is nearly zero, suggesting a negligible bias between the two techniques. The majority of the data points fall within the agreement limits, indicating that the differences between the measurements obtained by both instruments are consistent and within an acceptable range. However, a few data points (with high permeability values) are outside the agreement limits, indicating possible outliers or increased variability in those particular measurements. This suggests that while the two techniques are generally in agreement, they may diverge at high permeability values.

#### Statistical comparison of permeability: TinyPerm 3 vs AutoScan

Table 1 presents some descriptive statistics for the four measurement datasets, namely, TinyPerm 3 in the host rock matrix, AutoScan in the host rock matrix, TinyPerm 3 in the burrow filling, and AutoScan in the burrow filling. The two measurements show similar statistics in the host rock matrix. On the other hand, the measurements exhibited greater variability in their statistics for burrow filling.

An ANOVA test was applied to determine if statistically significant differences existed in the permeability measurements of the host rock matrix and burrow fillings obtained using TinyPerm 3 and AutoScan. The null hypothesis stated that: ’There is a statistically significant difference between the permeability measurements of TinyPerm 3 and AutoScan’. The results, presented in Table 2 and 3, show a P value of 0.29 for the host rock matrix data and 0.46 for the burrow fillings data. These P values, which are above the typical threshold of 0.05, lead to the rejection of the null hypothesis, suggesting that there is no statistically significant difference in the permeability measurements conducted on the host rock matrix and burrow fillings using both TinyPerm 3 and AutoScan.

Similarly, the statistical analysis conducted to examine the correlation between the permeability measurements obtained using TinyPerm 3 (as the dependent variable) and those obtained using AutoScan (as the independent variable) revealed P values less than 0.05. This analysis was based on the null hypothesis that there is no correlation between the independent and dependent variables. The P values, which were lower than 0.05, allowed us to reject this null hypothesis, leading to the conclusion that the permeability measurements obtained by both methods are significantly correlated. The strength of this correlation is underscored by an R$^2$ greater than 0.9 for permeability in both the host rock matrix and burrow fillings.

**Table 1:** Descriptive statistics of the TinyPerm 3 and AutoScan permeability measurements in the host rock matrix (HRM) and burrow filling (BF).

Statistic	HRM-TinyPerm 3	HRM-AutoScan	BF-TinyPerm 3	BF-AutoScan
Number of observations	32	32	32	32
Minimum	0.20	0.26	2.23	2.65
Maximum	3.82	4.73	1403.93	796.30
1st Quartile	0.92	1.04	11.70	12.17
Median	1.08	1.29	22.61	23.85
3rd Quartile	1.34	1.58	46.97	50.14
Mean	1.23	1.42	133.86	87.27
Variance (n-1)	0.42	0.62	94595.39	28980.07
Standard deviation (n-1)	0.65	0.79	307.56	170.24

**Table 2:** ANOVA test of the TinyPerm 3 and AutoScan permeability measurements in the host rock matrix (HRM).

Summary						
Groups	Count	Sum	Average	Variance		
HRM-TinyPerm 3	32	39.28	1.23	0.42		
HRM-AutoScan	32	45.43	1.42	0.62		

ANOVA						
Source of variation	*SS*	*df*	*MS*	*F*	*P-value*	*F crit*
Between groups	0.59	1	0.59	1.13	0.29	4.00
Within groups	32.48	62	0.52			
Total	33.08	63

**Table 3:** ANOVA test of the TinyPerm 3 and AutoScan permeability measurements in the Burrow Fill (BF).

Summary						
Groups	Count	Sum	Average	Variance		
HRM-TinyPerm 3	32	4283.53	133.86	94595.39		
HRM-AutoScan	32	2792.64	87.27	28980.07		

ANOVA						
Source of variation	*SS*	*df*	*MS*	*F*	*P-value*	*F crit*
Between groups	34730.89	1	34730.89	0.56	0.46	4.00
Within groups	3830839	62	61787.73			
Total	3865570.08	63				

## Discussion

The results of this study contribute significant insights into the challenging task of permeability measurement in bioturbated rocks, specifically focusing on the comparison between traditional AutoScan permeameter and the TinyPerm 3 instrument. This approach provides a fair understanding of the strengths and limitations of the TinyPerm 3 instrument. The agreement observed in the permeability measurements of the host rock matrix indicates the potential reliability of the handheld device, with both linear-linear and log-log plots demonstrating a close correlation between the two methods. The Bland-Altman plot for the host rock matrix further supports this, suggesting minimal bias with some divergence at higher permeabilities, underscoring the importance of understanding potential limitations, especially in high-permeability scenarios.

In the context of burrow filling permeability, the study revealed a bimodal distribution, emphasizing the variability in texture and pore type within the samples. The agreement in low-permeability measurements is notable, reaffirming the efficacy of the TinyPerm 3 instrument. However, a slight overestimation by TinyPerm 3 in high-permeability measurements is identified through both graphical representation and quantitative analysis. The Bland-Altman plot for burrow filling echoes the trends observed in host rock matrix measurements, indicating general agreement but potential divergence at higher permeabilities. These findings highlight the importance of considering instrument accuracy across a broad range of permeabilities, particularly in reservoirs where the burrow fill can exhibit significant variations.

The study suggested that the portable handheld device (TinyPerm 3) accurately captures low-permeability values, demonstrating its precision in such scenarios. The consistent higher readings by TinyPerm 3 in high-permeability measurements, particularly when AutoScan values exceed 300 mD, should be further investigated. TinyPerm was reported to record higher permeability values than other methods^35,36^, which could be attributed to the lower contact pressure of TinyPerm 3. Filomena et al.^36^ derived empirical equations to correlate different permeability devices including TinyPerm 2 (the previous edition of TinyPerm system).

The strong agreement between TinyPerm 3 and AutoScan measurements has significant implications for real-time field applications. Portable permeameters like the TinyPerm 3 enable geologists and reservoir engineers to make rapid, in-situ permeability assessments during field campaigns. This capability supports immediate decision-making, such as optimizing sampling strategies and identifying high-permeability zones for further investigation. Additionally, the ability to collect numerous non-destructive measurements quickly allows for more detailed spatial mapping of permeability variations within outcrops or cores, which can lead to more accurate reservoir models and improved production strategies.

The portability of handheld permeameters is particularly valuable when dealing with bioturbated rocks, which present several practical challenges. One major issue is the need to collect large, heavy rock samples to obtain a representative element volume (REV) for accurate permeability characterization, as shown by Eltom et al.^30^ Another challenge is the impact of natural surface weathering on outcrop measurements, where alteration can extend several inches below the surface, especially in weakly cemented bioturbated rocks, as highlighted by Dinwiddie et al.^37^ In such cases, traditional methods require drilling vertical or horizontal cores to access unweathered material. A handheld permeameter, however, enables on-site measurements with minimal surface preparation-for example, by drilling a small hole at a fresh road cut or accessible cliff face. Furthermore, in friable and weakly cemented strata, obtaining intact samples for laboratory analysis can be difficult, making in-situ measurement approaches even more advantageous.

The portable handheld permeameter becomes particularly advantageous, offering a practical solution for permeability assessments in such challenging geological settings. Last, the convenience of in situ field measurements with handheld permeameters on roadcuts or cliffs eliminates the need to correct laboratory samples for overburden issues, where previous research has emphasized the challenges of obtaining representative samples for laboratory analysis. Even in the laboratory, portable handheld permeameters can eliminate the need to prepare a flat rock surface by slabbing cores or cutting rock boulders because they can tolerate rough surfaces.

The implications of these results are substantial for both field and laboratory practices. With its portability, ease of use and real-time data recording capabilities, the handheld permeameter offers a valuable tool for on-the-spot permeability measurements. In particular, the field measurement context can inform and enhance the sampling process, especially in the case of large rock samples that may not fit into standard laboratory instruments or very fragile rocks. With its real-time capability, the portable handheld permeameter encourages researchers to perform more permeability measurements in the field. Ultimately, this work contributes to ongoing efforts to enhance the accuracy of permeability assessments in bioturbated rocks. By measuring permeability at the host rock matrix and burrow fill spots on large rock samples using a portable handheld permeameter in the field combined with the right averaging technique^38^, a better estimate of bioturbated rock permeability can be obtained in a shorter time and with minimal effort. These better permeability estimates are crucial for effective reservoir management and characterization^39^ by providing more realistic permeability values of bioturbated strata to reservoir simulators.

Although this study focuses on a single formation, The Hanifa Formation’s characteristics make it an ideal candidate for this comparative study. Its well-documented bioturbated strata^34^ exhibit a range of permeability values within the host rock matrix and burrow fills, allowing for a comprehensive assessment of both the TinyPerm 3 and AutoScan systems across varying permeability ranges. This internal variability within a single formation provides a robust dataset for evaluating the performance of portable permeameters in heterogeneous carbonate reservoirs. Moreover, the Hanifa Formation is representative of many *Thalassinoides*-bioturbated hydrocarbon reservoirs worldwide, such as the Upper Devonian Wabamun Group in Alberta^15^, the Arab-D reservoirs in the Middle East^6,40^, and the Cretaceous reservoirs of Saudi Arabia^41^. Other examples include the Ordovician carbonate reservoir in the Tarim Basin, China^42^, the Ordovician Bighorn Dolomite in Wyoming, USA^1^, the Devonian Palliser Formation^12^, the Cambrian Zhushadong and Longwangmiao Formations in China^43,44^, and the early-middle Miocene strata in Kuwait^45^. This enhances the potential applicability of our findings to similar geological settings in the oil and gas industry. However, we acknowledge that expanding this study to include other formations with different bioturbation styles, lithologies, and depositional environments would further validate the broader applicability of portable permeameters in diverse geological contexts.

This study opens up several avenues for future research. First, investigating the performance of portable permeameters across a wider range of bioturbated formations could further validate their broad applicability. Second, integrating portable permeameter data with other field measurements (e.g., portable gamma-ray logs) could provide a more comprehensive understanding of rock characteristics. Lastly, exploring the use of machine learning algorithms to estimate permeability from rock images or interpret portable permeameter data in real-time could significantly enhance field-based reservoir characterization efforts.

## Novelty of the research

The novelty of this research lies in its comparative approach to evaluating the effectiveness of a portable permeability measurement device (TinyPerm 3) against a traditional laboratory method (AutoScan) in bioturbated strata. This study not only demonstrates the feasibility of using portable devices for in situ measurements but also provides empirical evidence supporting their reliability and accuracy. The findings have important implications for field geologists and reservoir engineers, offering a practical and efficient solution to the persistent challenges of measuring permeability in complex geological settings such as bioturbated carbonate rocks.

## Conclusions

In conclusion, this research presents a comparative analysis of two permeability measurement techniques for bioturbated carbonate rocks comparing the portable handheld TinyPerm 3 permeameter with the laboratory AutoScan spot permeameter. Using both graphical and statistical methods, this comparative study shows a good agreement between the two techniques. It highlights the strengths and limitations of each method offering valuable insights for researchers and reservoir simulators. This detailed one-to-one comparison and evaluation have not been extensively explored in previous studies. Following are the main findings of this work:

- The portable TinyPerm 3 permeameter shows good agreement with the laboratory AutoScan system for measuring permeability particularly in the low-permeability range of bioturbated rock samples, offering a new reliable alternative to traditional laboratory methods.
- TinyPerm 3 consistently records higher permeability values in highly permeable sections of bioturbated rocks (greater than 300 mD) indicating difference in measurement sensitivity and accuracy at very high values.
- The accuracy and portability of the TinyPerm 3 permeameter addresses key challenges in sampling bioturbated rocks, such as the logistical difficulties of handling large rock samples and the need of sample preparation. This represents a substantial advancement in field permeability assessments.
- Utilizing TinyPerm 3 in the field enables a greater number of in situ permeability data points, leading to a more precise characterization of bioturbated rocks.

Overall, by integrating portable technology into permeability assessment, this research contributes to more effective and practical permeability measurement offering practical solutions for field geologists and reservoir engineers when dealing with the complex geological settings of bioturbated rocks where traditional laboratory methods are often impractical. Ultimately this will result in a more informed reservoir characterization and management practices.

## Data Availability

The data that support the findings of this study are available from the corresponding author upon reasonable request. For inquiries, please contact Ayman Al-Lehyani at Allehyani@kfupm.edu.sa.

## Abbreviations

ANOVA: Analysis of variance
BF: Burrow fill
D-PermFFM: Dual-permeability fluid flow media
D-PorFFM: Dual-porosity fluid flow media
HRM: Host rock matrix
HST: Highstand systems tract
mD: Millidarcy
NER: New England research
REV: Representative element volume
